# Visible-Light-Promoted Transition-Metal-Free Construction of 3-Perfluoroalkylated Thioflavones

**DOI:** 10.3389/fchem.2022.953978

**Published:** 2022-07-13

**Authors:** Chunhua Ma, Hui Meng, Xing He, Yuqin Jiang, Bing Yu

**Affiliations:** ^1^ Collaborative Innovation Centre of Henan Province for Green Manufacturing of Fine Chemicals, Key Laboratory of Green Chemical Media and Reactions, Ministry of Education, Henan Engineering Research Centre of Chiral Hydroxyl Pharmaceutical, Henan Engineering Laboratory of Chemical Pharmaceutical and Biomedical Materials, School of Chemistry and Chemical Engineering, Henan Normal University, Xinxiang, China; ^2^ Green Catalysis Center, College of Chemistry, Zhengzhou University, Zhengzhou, China

**Keywords:** photocatalysis, perfluoroalkylation, cyclization, thioflavone, antitumor

## Abstract

A visible-light-promoted transition-metal-free perfluoroalkylation/cyclization reaction was developed with 9-mesityl-10-methylacridinium perchlorate (Acr^+^-Mes·ClO_4_^−^) as the photocatalyst, by which various perfluoroalkyl-substituted heterocycles including thioflavones, oxindoles, and quinoline-2,4(1*H*,3*H*)-diones were prepared at room temperature. Moreover, the potential of this sustainable method is demonstrated by the excellent *in vitro* anti-lymphoma and cervical carcinoma activity of the novel 3-perfluoroalkylated thioflavone **3m**.

## Introduction

Thioflavone is a privileged scaffold that is ubiquitous in natural products, bioactive molecules, and functional materials (Dong et al., 2018). The derivatives of thioflavones have been found to exhibit intriguing biological activities, such as anticancer, (Wang et al., 1996), and anti-malarial (Razdan et al., 1978). Consequently, the construction of thioflavones with various substituents has attracted considerable attention (Kumar and Bodas, 2001; Pan et al., 2018; Sangeetha and Sekar, 2019; Yang et al., 2020; Zheng et al., 2020; Wang W. et al., 2021; Lee Jae, 2021). The perfluoroalkyl group, especially trifluoromethyl group, is one of the most prominent substituents in medicinal chemistry, which is essential for more than 70 approved drugs (Schiesser et al., 2020). It may be attributed to the fact that perfluoroalkyl group can remarkablely improve the pharmacokinetics properties, lipophilicity and target inhibitory of the parent compounds (Müller et al., 2007; Tang et al., 2015; Tang et al., 2017a; Tang et al., 2017b; Wang et al., 2019; Ma et al., 2021a; Chen et al., 2022). In this context, developing the method to access perfluoroalkyl containing thioflavone is of great significance. However, there are currently no available reports to deliver this fragment.

**Scheme 1:**
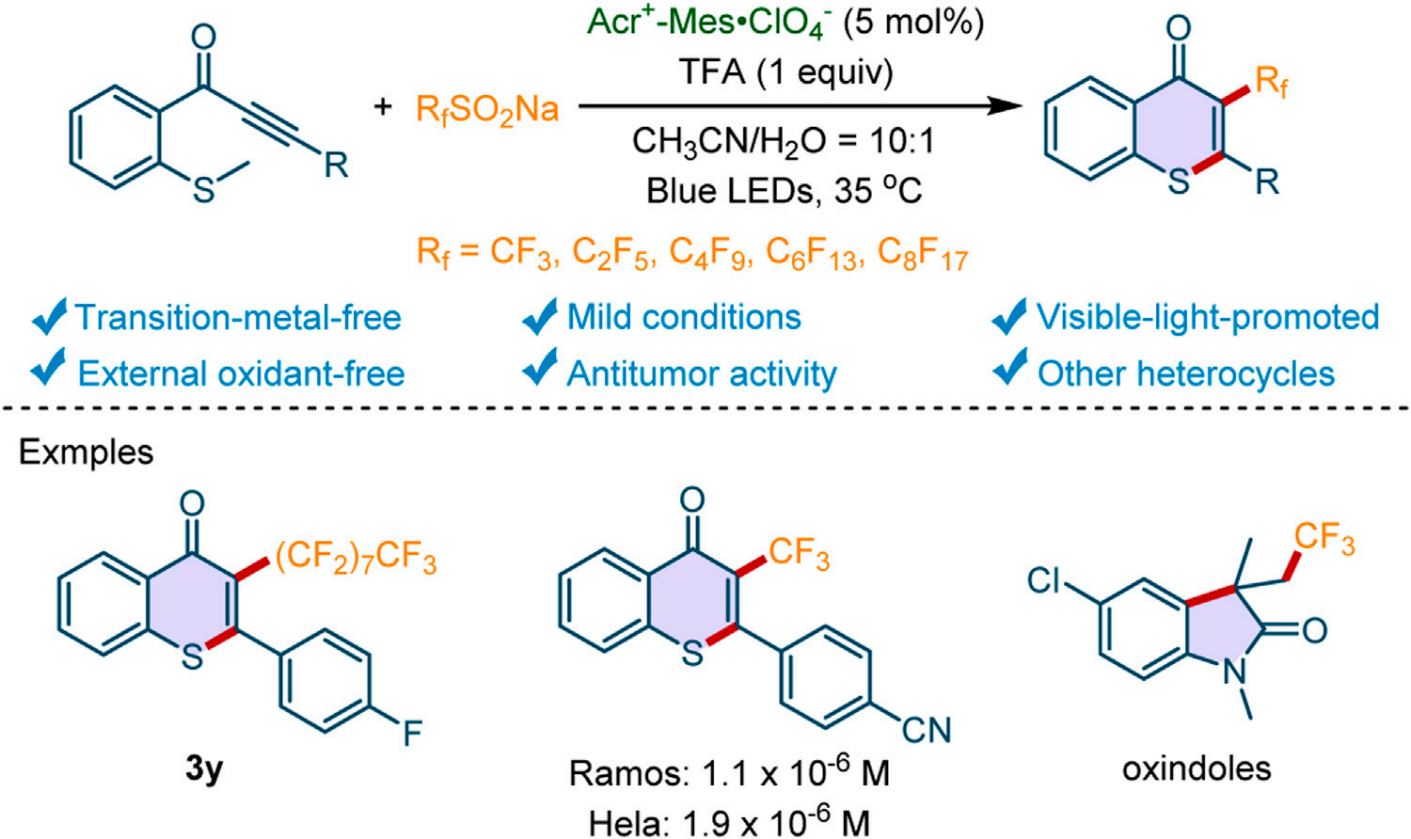
Transition-metal-free photocatalytic perfluoroalkylation/cyclization reactions.

Recently, methylthiolated alkynone is used as a unique starting material to access thioflavones containing diverse substitutes by the radical-initiated cyclization (Zhou et al., 2006; Alcaide et al., 2017). For instance, Song and coworkers developed a highly efficient approach to synthesizing phosphoryl-, acyl-, and sulfenyl-containing thioflavones from methylthiolated alkynones (Xu et al., 2019). Huang’s group realized the reaction of AgSCF_3_ with methylthiolated alkynones for the synthesis of 3-trifluoromethylthiolated thioflavones with (NH_4_)_2_S_2_O_8_ as the oxidant at 80°C (Wang L. et al., 2021). Du and Zhao *et al* reported the synthesis of selenyl/sulfenyl thioflavones with phenyliodine(III) bis(trifluoroacetate) (PIFA) as an oxidant (Ai et al., 2020). Recently, Ye and Wu’s group realized an Ir-photocatalyzed radical relay reaction of methylthiolated alkynones and potassium metabisulfite in the presence of sodium methylsulfinate (Liu et al., 2022). With the radical-initiated cyclization of methylthiolated alkynone, our group has developed some protocols to access the phosphorylated, (Liu et al., 2020), acylated, (Zhu et al., 2021), sulfonylated, (Feng et al., 2020; Jiang et al., 2020), and thiocyanated thioflavones (Zeng et al., 2021). Nevertheless, the synthesis of thioflavones bearing perfluoroalkyl-substituents is rarely reported. Considering the critical roles of F-containing groups in the development of bioactive molecules, we herein disclosed that perfluoroalkyl-containing thioflavones could be accessed through photocatalytic cascade perfluoroalkylation/cyclization reactions in the presence of sodium perfluoroalkanesulfinates (R_f_SO_2_Na) as the perfluoroalkyl source (R_f_ = CF_3_, C_2_F_5_, C_4_F_9_, C_6_F_13_, C_8_F_17_) and 9-mesityl-10-methylacridinium perchlorate (Acr^+^-Mes·ClO_4_^−^) as a transition-metal-free photocatalyst (Scheme 1). More importantly, the synthesized compounds exhibited excellent *in vitro* antitumor properties, which indicated that the unique protocol could be used to deliver novel antitumor hit compounds.

## Results and Discussion

We chose methylthiolated alkynone (**1a**) and CF_3_SO_2_Na (**2a**) as model substrates to investigate the perfluoroalkylation/cyclization reaction in CH_3_CN under 18 W blue LEDs irradiation at 35°C. Fortunately, when Acr^+^-Mes·ClO_4_^−^ was used as the photocatalyst and HCl as the acid additive, **1a** and **2a** could be converted into the corresponding trifluoromethylated thioflavone **3a** in 37% yield (Table 1, entry 1). Evaluation of the different photocatalysts showed that Acr^+^-Mes·ClO_4_^−^ was the best photocatalyst for this process (Table 1, entries 2-4). Different acidic additives were screened to further improve the efficiency of this transformation (Table 1, entries 5-8). The experimental results indicated that trifluoroacetic acid (TFA) showed the highest reactivity, affording **3a** in 44% yield (Table 1, entry 7). To further improve the yield, a range of solvents, including DCM, DCE, CHCl_3_, 1,4-dioxane, DMF, DMSO, EtOH, H_2_O, and acetonitrile aqueous were evaluated (Table 1, entries 9-20). The mixed solvent CH_3_CN/H_2_O (v/v = 10:1) was found to be the optimal solvent system, and the desired product **3a** could be obtained in 56% yield (Table 1, entry 19). Furthermore, increasing the amount of **2a** to 3 equiv afforded the highest yield (Table 1, entry 21). The control experiments confirmed the photochemical nature of this transformation, as no product was observed in the absence of photocatalyst or visible light (Table 1, entries 22-23). The reaction efficiency was decreased to 35% in the absence of TFA, which indicated that the acid plays an important role in promoting the transformation (Table 1, entry 24). Taken together, the optimal reaction conditions were established as follows: **1a** (0.2 mmol), **2a** (3 equiv), Acr^+^-Mes·ClO_4_^−^ (5 mol%) as catalyst, TFA (1 equiv) as additive, CH_3_CN/H_2_O (v/v = 10:1) as solvent, at 35°C under the irradiation of blue LEDs (*λ*_max_ = 460 nm) for 5 h.

**TABLE 1:** Optimization of reaction conditions^a^.

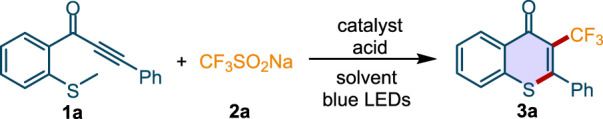				
**Entry**	**Catalyst (5 mol%)**	**Acid (1 equiv)**	**Solvent**	**Yield (%)^b^**
1	Acr^+^-Mes·ClO_4_^–^	HCl	MeCN	37
2	Ru(bpy)_3_Cl_2_	HCl	MeCN	13
3	**PC3**	HCl	MeCN	24
4	**PC4**	HCl	MeCN	N. R.
5	Acr^+^-Mes·ClO_4_^–^	H_2_SO_4_	MeCN	22
6	Acr^+^-Mes·ClO_4_^–^	AcOH	MeCN	29
7	Acr^+^-Mes·ClO_4_^–^	TFA	MeCN	44
8	Acr^+^-Mes·ClO_4_^–^	Pivalic acid	MeCN	39
9	Acr^+^-Mes·ClO_4_^–^	TFA	DCM	43
10	Acr^+^-Mes·ClO_4_^–^	TFA	DCE	37
11	Acr^+^-Mes·ClO_4_^–^	TFA	CHCl_3_	37
12	Acr^+^-Mes·ClO_4_^–^	TFA	Dioxane	trace
13	Acr^+^-Mes·ClO_4_^–^	TFA	DMF	trace
14	Acr^+^-Mes·ClO_4_^–^	TFA	DMSO	trace
15	Acr^+^-Mes·ClO_4_^–^	TFA	EtOH	trace
16	Acr^+^-Mes·ClO_4_^–^	TFA	H_2_O	trace
17	Acr^+^-Mes·ClO_4_^–^	TFA	MeCN/H_2_O = 5:1	41
18	Acr^+^-Mes·ClO_4_^–^	TFA	MeCN/H_2_O = 8:1	53
19	Acr^+^-Mes·ClO_4_^–^	TFA	MeCN/H_2_O = 10:1	56
20	Acr^+^-Mes·ClO_4_^–^	TFA	MeCN/H_2_O = 15:1	53
21^c^	Acr^+^-Mes·ClO_4_^–^	TFA	MeCN/H_2_O = 10:1	75
22^c^	–	TFA	MeCN/H_2_O = 10:1	N. R.
23^c,d^	Acr^+^-Mes·ClO_4_^–^	TFA	MeCN/H_2_O = 10:1	N. R.
24^c^	Acr^+^-Mes·ClO_4_^–^	–	MeCN/H_2_O = 10:1	35

a Reaction conditions: **1a** (0.2 mmol), **2a** (2 equiv), photocatalyst (5 mol%), acid (1 equiv), solvent (3 ml), 35^o^C, blue LEDs, 5 h under air atmosphere. **PC3**, 2,4,6-triphenylpyrylium tetrafluoroborate; **PC4**, 10-(3,5-dimethoxyphenyl)-9-mesityl-1,3,6,8-tetramethoxyacridin-10-ium tetrafluoroborate.

b Isolated yields. N. R., No reaction.

c **2a** (3 equiv).

d Without light.

**Scheme 2:**
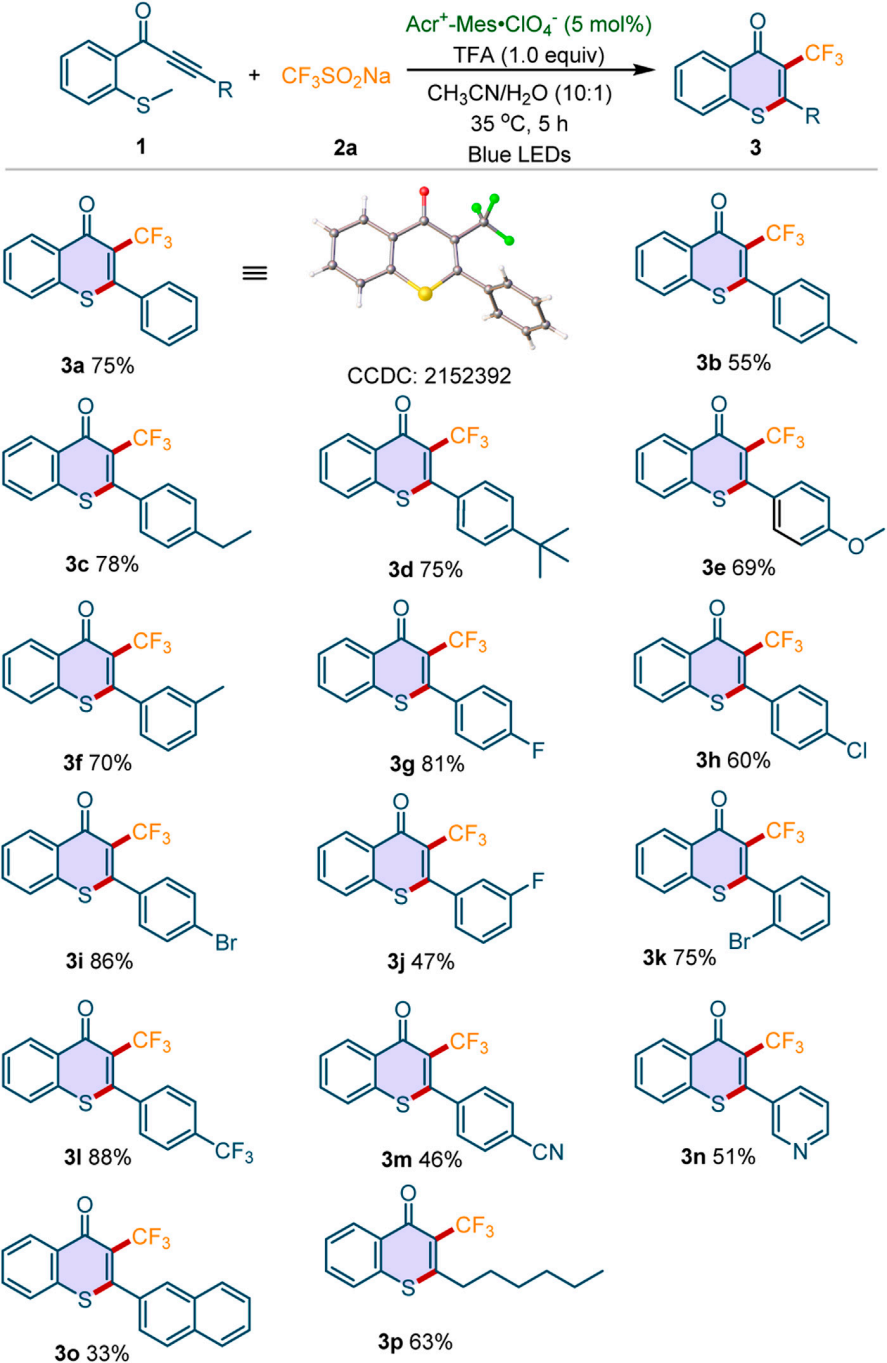
Scope of methylthiolated alkynones. Reaction conditions: **1** (0.2 mmol), **2a** (3 equiv), Acr^+-^Mes·ClO_4_^−^ (5 mol%), TFA (1 equiv), CH_3_CN/H_2_O (10:1, 3 ml), 35^o^C, blue LEDs, 5 h under air atmosphere. Isolated yields were given.

With the optimal conditions in hand, we further explored the scope and generality of this cascade perfluoroalkylation/cyclization reaction. Firstly, the reactivities of electron-rich or electron-deficient methylthiolated alkynones were investigated. As depicted in Scheme 2, the electron-donating groups (4-Me, 4-Et, 4-^*t*^Bu, 4-MeO, 3-Me) were well tolerant, giving the desired products **3b**-**3f** in 55–78% yields. For the substrates with electron-withdrawing groups, we found that both halogen substitutions (4-F, 4-Cl, 4-Br, 3-F, 2-Br) and CF_3_ group were compatible with this transformation, and the corresponding products **3g**-**3l** were obtained in moderate to good yields. Moreover, the methylthiolated alkynone containing a strong electron-withdrawing substituent (4-CN) worked well under the standard conditions (**3m**). The pyridine or naphthalene analogs (**1n**-**1o**) were also found to be tolerant to the optimized condition. To our delight, the substrate containing alkyl group instead of aryl ring is well tolerant, affording the desired product **3p** in 63% yield. Moreover, the structure of **3a** was unambiguously confirmed by X-ray crystallographic analysis.

**Scheme 3:**
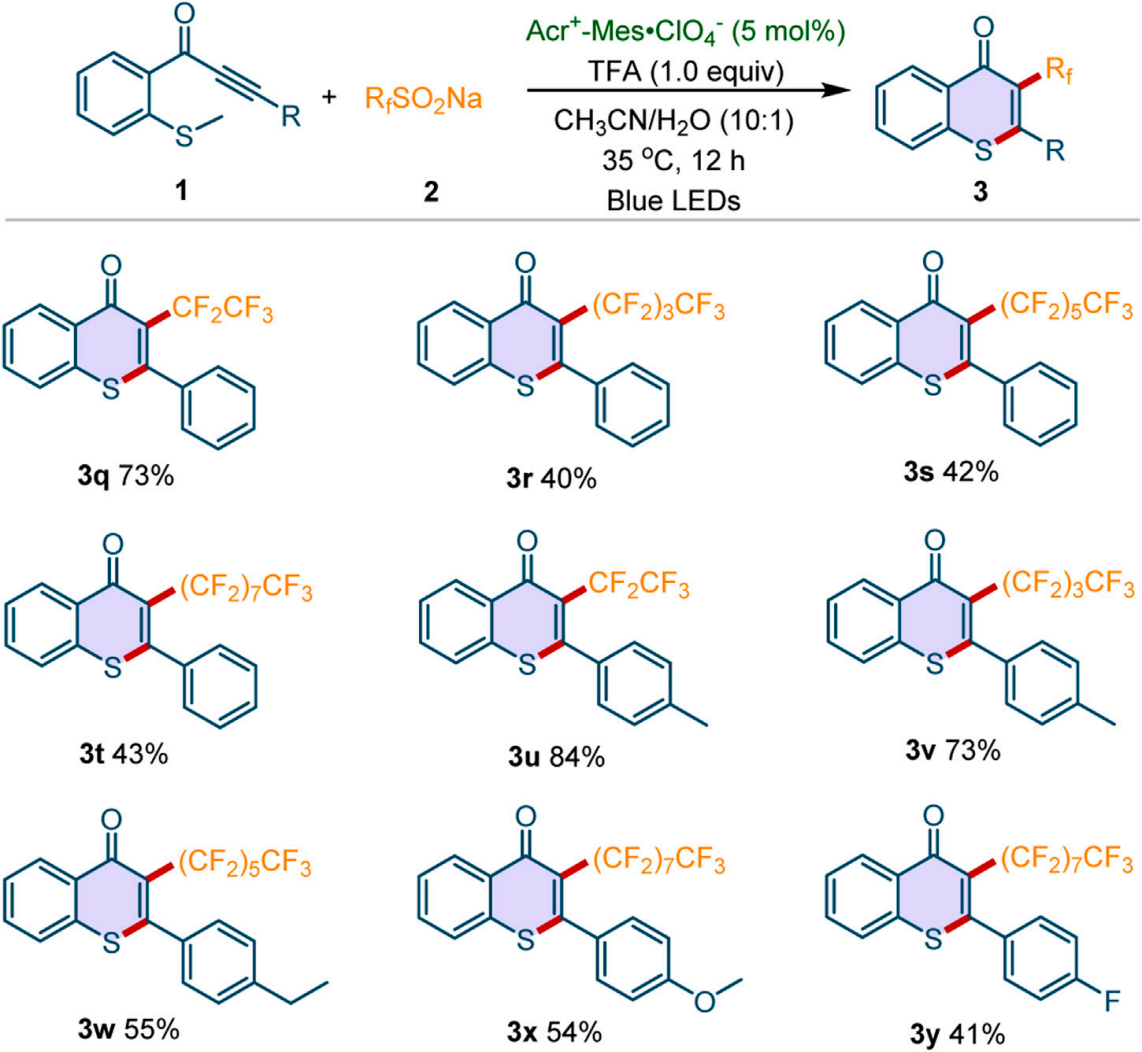
Scope of sodium perfluoroalkanesulfinates. Reaction conditions: **1** (0.2 mmol), **2a** (3 equiv), Acr^+^-Mes·ClO_4_^−^ (5 mol%), TFA (1 equiv), CH_3_CN/H_2_O (v/v = 10:1, 3 mL), 35^o^C, blue LEDs, 12 h under air atmosphere. Isolated yields were given.

Subsequently, the scope of sodium perfluoroalkanesulfinates **2** was examined. As shown in Scheme 3, a variety of sodium perfluoroalkanesulfinates were well tolerated in this protocol. For example, CF_3_CF_2_SO_2_Na, CF_3_(CF_2_)_3_SO_2_Na, CF_3_(CF_2_)_5_SO_2_Na and CF_3_(CF_2_)_7_SO_2_Na reacted well with **1a**, furnishing the perfluoroalkyl-substituted thioflavones **3q**-**3t** in moderate to good yields. Meanwhile, the sodium perfluoroalkanesulfinates reacted well with methylthiolated alkynones **1** bearing different substituents (Me, Et, OMe, F), affording the desired products **3u**-**3y** in 41–84% yields. Notably, all the synthesized 3-perfluoroalkylated thioflavones are new compounds.

**Scheme 4:**
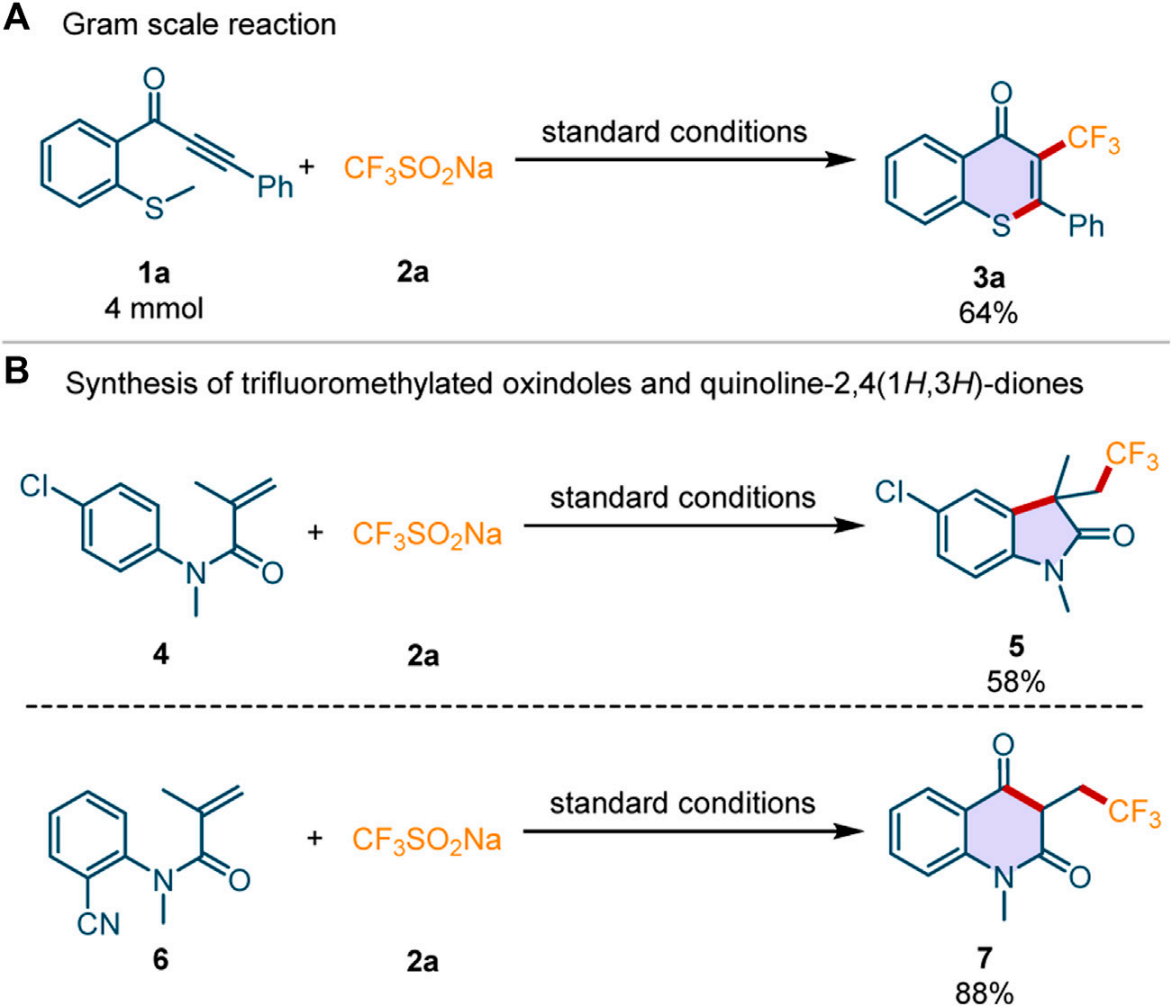
The gram-scale synthesis and the application of this perfluoroalkylation/cyclization reactions.

To evaluate the applicability of this perfluoroalkylation/cyclization reaction in the pharmaceutical industry, it was scaled up to 4 mmol under standard conditions. Delightfully, the desired product **3a** was obtained in 64% yield (Scheme 4A), which indicates that the transformation may be used in drug development. Inspired by the successful usage of this photocatalytic system in the synthesis of perfluoroalkylated thioflavones, we then applied the sustainable system in the construction of other perfluoroalkylated heterocycles under the standard conditions (Scheme 4B). The substrates *N*-(4-chlorophenyl)-*N*-methylmethacrylamide **4** and *N*-(2-cyanophenyl)-*N*-methylmethacrylamide **6** could be converted into the corresponding trifluoromethylated oxindole **5** and trifluoromethylated quinoline-2,4(1*H*,3*H*)-dione **7** in 58 and 88% yields, respectively.

**Scheme 5:**
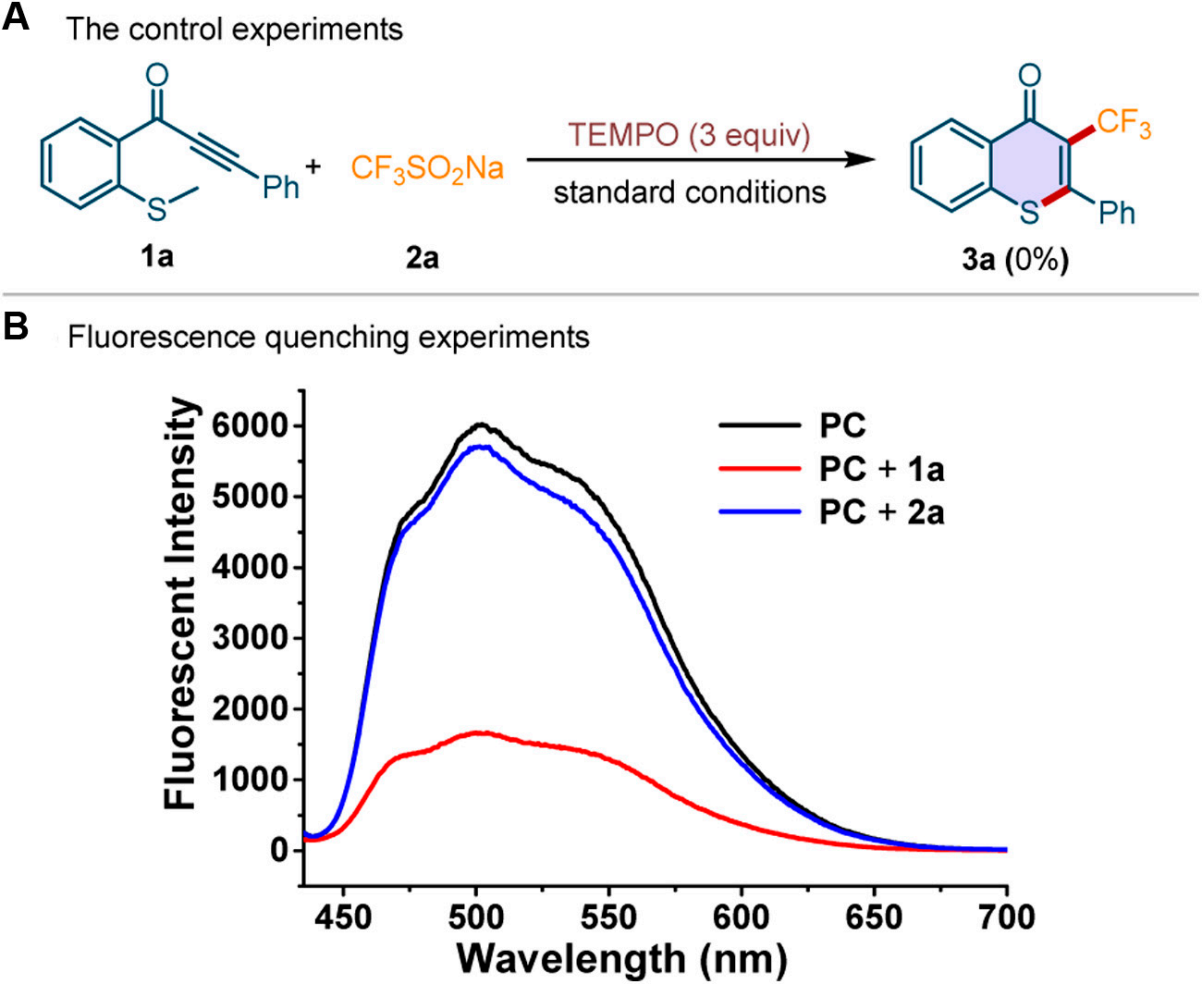
The control experiment and fluorescence quenching experiment.

To explore the reaction mechanism, the control experiment and the Stern–Volmer fluorescence quenching experiments were performed (Scheme 5). The addition of radical quencher, 2,2,6,6-tetramethylpiperidinyl-1-oxyl (TEMPO), to the standard conditions completely prevented the reaction (Scheme 5A). It indicated that a radical pathway may be involved in this photocatalytic transformation. We conducted the Stern–Volmer fluorescence quenching experiment by mixing the photocatalyst Acr^+^-Mes·ClO_4_^−^ (**PC**) with methylthiolated alkynone **1a** and CF_3_SO_2_Na **2a**, respectively. The results were depicted in Scheme 5B. The luminescence effect was obviously quenched by the addition of **1a**, while it is hardly changed by the addition of **2a**. Moreover, a strong linear relationship was observed between I_0_/I and the concentration of **1a**, indicating that **1a** could act as an available quencher of the excited state of the photocatalyst (for details see the Supplementary Material).

**Scheme 6:**
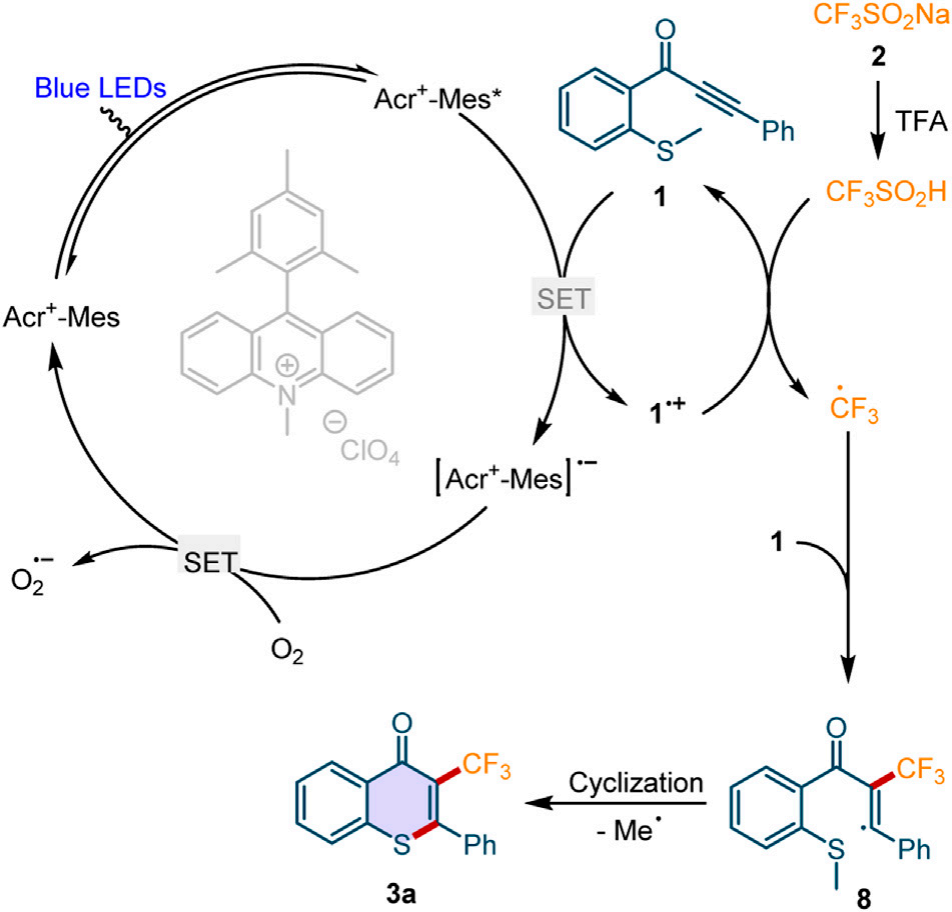
The proposed mechanism.

Based on the above experimental results and the previous reports, (Neogi et al., 2020; Ma et al., 2021b; Yang et al., 2021; Ma et al., 2022; Shen et al., 2022; Zhu et al., 2022), we proposed a plausible reaction mechanism for this photocatalyzed perfluoroalkylation/cyclization reaction (Scheme 6). Under the visible light irradiation, Acr^+^-Mes was activated into the excited state Acr^+^-Mes*, which then oxidized the substrate **1** to afford the radical cation **1** ^•+^ and the radical anion [Acr^+^-Mes]^•-^. CF_3_SO_2_Na **2a** was *in situ* converted into CF_3_SO_2_H in the presence of the acid TFA. Then the radical cation **1** ^•+^ reacted with CF_3_SO_2_H *via* a single-electron transfer (SET) process to generate the CF_3_ radical and regenerate the substrate **1**. The desired product **3** was afforded by the addition of CF_3_ radical to the triple bond of **1** and a subsequent intramolecular demethylation cyclization. On the other hand, the [Acr^+^-Mes]^•-^ could be oxidized by the O_2_ in the air to regenerate the ground state of the photocatalyst and complete the photoredox cycle.

**Scheme 7:**
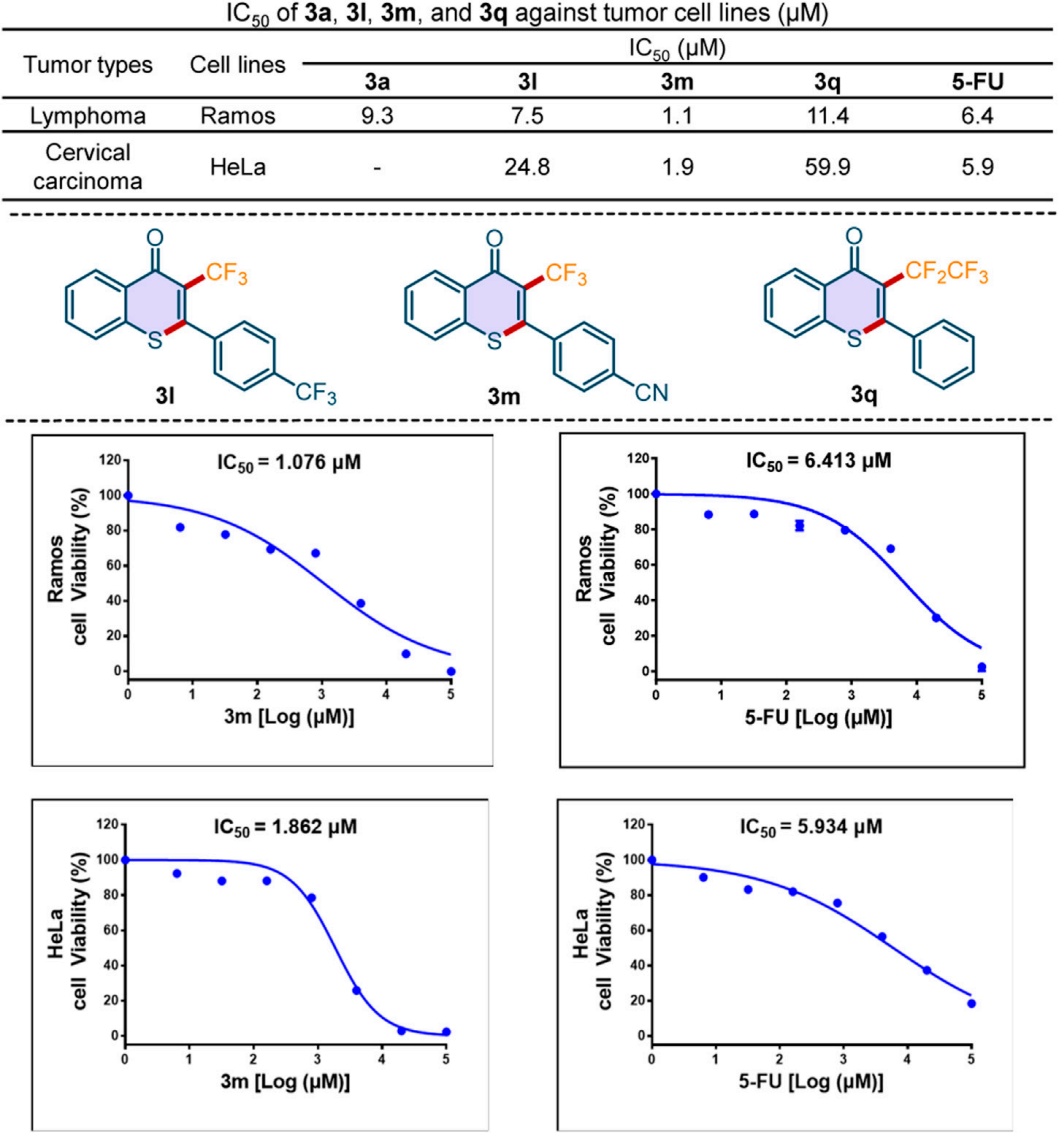
The antitumor activity of the representative compounds.

To highlight this sustainable method in drug development, we evaluated the *in vitro* antitumor activity of the novel 3-perfluoroalkylated thioflavones. As shown in Scheme 7, compound **3m** exhibited better antitumor activities against Ramos cell and Hela cell than that of broad-spectrum antitumor drug 5-fluorouracil (**5-FU**), which indicated that our compound has the potential to treat human B cell lymphoma and human cervical carcinoma. Because tumor resistance to chemotherapeutic drugs is a significant issue in the clinical treatment of carcinoma, the development of novel chemical entries, such as the 3-perfluoroalkylated thioflavones, is of great value.

## Conclusion

In summary, we have developed a visible-light-induced perfluoroalkylation/cyclization of methylthiolated alkynones for the mild and rapid construction of 3-perfluoroalkylated thioflavones. It has been demonstrated that this radical involved strategy is tolerant of a variety of functional groups and could be applied to the construction of other perfluoroalkylated heterocycles, such as oxindoles and quinoline-2.4(1*H*,3*H*)-diones. Moreover, compound **3m** exhibited robust antitumor activity, which provides a novel chemical entry for the clinical treatment of human lymphoma and cervical carcinoma. The structural modification of these novel compounds is currently underway in our laboratory.

## Data Availability

The original contributions presented in the study are included in the article/Supplementary Material, further inquiries can be directed to the corresponding authors.
