# Supplementary material for: Visible-Light-Promoted Transition-Metal-Free Construction of 3-Perfluoroalkylated Thioflavones
Source: Front Chem. 2022 Jul 13;10:953978. doi: 10.3389/fchem.2022.953978 (PMC9326344; doi:10.3389/fchem.2022.953978)
Supplement: Supplementary file 2 [file DataSheet2.docx]

***Supporting Information***

**Table of Contents**

**1. General information……………...………………….………………....……S2**

**2. Experimental procedures…………………………………………………....S2**

**3. Characterization of compounds…………………………………………......S6**

**4. NMR copies of products……………………….………………..……….…..S17**

**5. X-ray crystallographic data for 3a…………….……………………………S57**

**6. References…………….………………………………………………………S58**

**1. General information**

**1.1 Materials and instruments**

Sodium trifluoromethanesulfinate (CF_3_SO_2_Na), Acr^+^-Mes·ClO_4_^–^ were purchased from Tansoole, Shanghai, China. Other reagents were purchased from Bidepharm.com. Unless otherwise stated, all commercially available reagents were directly used without further purification. All solvents were purified by standard methods prior to use. All reactions were monitored by thin layer chromatography (TLC), and column chromatography was carried out on 100-200 mesh of silica gel purchased from Damas-beta. All nuclear magnetic resonance (NMR) spectra were recorded on a Bruker Avance 600 MHz or a Bruker Avance 400 MHz in CDCl_3_ at room temperature (20 ± 3 °C), using tetramethylsilane as internal standard. High resolution mass spectra (HRMS) were conducted on a 3000-mass spectrometer, using Bruker compact Qq TOF MS/MS system with the ESI technique.

The photochemical reactions were carried out under visible light irradiation by blue LEDs at 35 ^o^C. The photo reaction system was equipped as the Figure S1. Eighteen 3W blue LEDs purchased from the Genesis Photonics were fixed into two radiator panels.


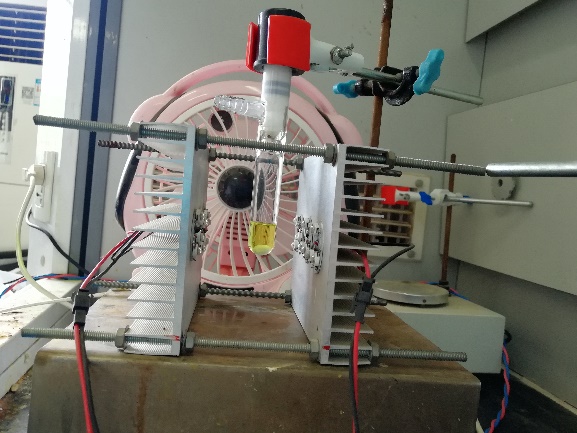

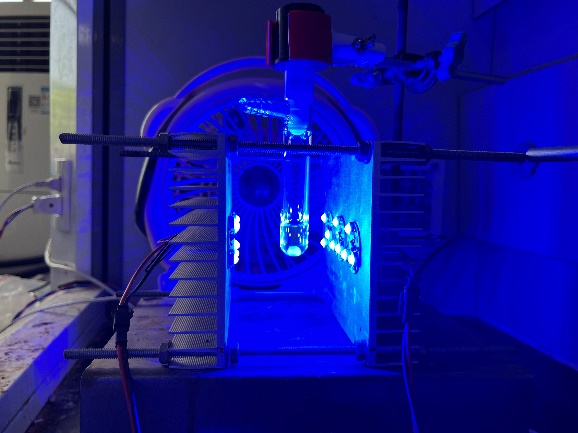


Figure S1. The photo reaction system.

1. **Experimental procedures**
   1. **General experimental procedures for the perfluoroalkylation/cyclization reactions**

In a 10 mL Schlenk reaction tube, methylthiolated alkynones **1** (0.2 mmol) and R_f_SO_2_Na **2** (3.0 equiv.) were added to the mixture solution of MeCN and H_2_O (10:1, 3 mL), and then TFA (1.0 equiv.), Acr^+^-Mes·ClO_4_^–^ (5 mol%) were added. The mixture was stirred at room temperature with Blue LEDs irradiation for 5 h under air. After the reaction was completed, the solvent was quenched with water (10 mL), and then ethyl acetate (15 mL) was added three times for extraction. The combined organic layers were dried over anhydrous Na_2_SO_4_. After filtered and evaporated under vacuum, the residue was purified by silica gel chromatography (petroleum ether/ethyl acetate = 60/1) to afford the desired product.

**The structure of photocatalysts**

**Control experiments**

In a 10 mL Schlenk reaction tube, 1-(2-(methylthio)phenyl)-3-phenylprop-2-yn-1-one **1a** (0.2 mmol) and CF_3_SO_2_Na **2a** (3.0 equiv.) were added to the mixture solution of MeCN and H_2_O (10:1, 3 mL), and then TFA (1.0 equiv.), Acr^+^-Mes·ClO_4_^–^ (5 mol%) were added. Afterward, TEMPO ((2,2,6,6-tetramethylpiperidin1-yl)oxidanyl) (3.0 equiv.) was added into the mixture. The mixture was allowed to stir at Blue LEDs irradiation for 5h in air. After the reaction was completed, the solvent was quenched with water (10 mL), and then ethyl acetate (15 mL) was added three times for extraction. The combined organic layers were dried over anhydrous Na_2_SO_4_. After filtered and evaporated under vacuum, **3a** was isolated by silica gel chromatography in 0% yield.

- 1. **Expand experiments**

In a 10 mL Schlenk reaction tube, N-(4-chlorophenyl)-N-methylmethacrylamide **4** (0.2 mmol) and **2a** (3.0 equiv.) were dissolved in MeCN : H_2_O =10:1 (3 mL) , and then TFA (1.0 equiv.), Acr^+^-Mes·ClO_4_^–^ (5 mol%) were added. The mixture was stirred at Blue LEDs for 8 h in air. After the reaction was completed, the solvent was quenched with water (10 mL), and then the ethyl acetate (15 mL) was added three times for extraction. The combined organic layers were dried over anhydrous Na_2_SO_4_. After filtered and evaporated under vacuum, the residue was purified by silica gel chromatography to afford the desired product **5** in 58% yield.

In a 10 mL Schlenk reaction tube, N-(2-cyanophenyl)-N-methylmethacrylamide **6** (0.2 mmol) and **2a** (3.0 equiv.) were dissolved in MeCN : H_2_O =10:1 (3 mL), and then TFA (1.0 equiv.), Acr^+^-Mes·ClO_4_^–^ (5 mol% equiv.) were added. The mixture was stirred at Blue LEDs for 8 h in air. After the reaction was completed, the solvent was quenched with water (10 mL), and then the ethyl acetate (15 mL) was added three times for extraction. The combined organic layers were dried over anhydrous Na_2_SO_4_. After filtered and evaporated under vacuum, the residue was purified by silica gel chromatography to afford the desired product **7** in 88% yield.

- 1. **Procedure for emission quenching experiment**

Stern-Volmer fluorescence quenching experiments were run by mixing freshly prepared solution of 2.5×10^-5^ M solution of Acr^+^-Mes·ClO_4_^–^ in dry MeCN with the appropriate amount of **1a** or **2a** in a screw-top quartz cuvette at room temperature. After degassing the sample with a stream of N_2_ for 10 minutes, the solutions were irradiated at 380 nm and fluorescence was measured from 435 nm to 700 nm. The emission of the sample was collected.

Figure S1. (A) The emission spectra of 2.5×10^-5^ M solution of Acr^+^-Mes·ClO_4_^–^ with 2×10^-4^ M **1a** or **2a** under 380 nm irradiating. (B) The emission spectra of 2.5×10^-5^ M solution of Acr^+^-Mes·ClO_4_^–^ with various concentrations of **1a** under 380 nm irradiating. (C) The linear relationship between I_0_/I (I_0_ and I are the fluorescence intensities before and after adding the various concentration of **1a** or **2a**, respectively) and the corresponding concentration under 380 nm irradiating.

1. **The *in vitro* antitumor assay**

The *in vitro* antitumor assay against Ramos cell was evaluated by the CellTiter-Glo (Promega, USA) assay. Ramos cell suspensions were diluted in a growth medium to desired density and added 95 µL to a 96-well plate. 5 µL of the tested compounds were added to the 96-well plate. Then the cell was incubated at 37 ℃, 5% CO_2_ for 72 h. Equilibrate the assay plate to room temperature before measurement. Add 20 µL of CellTiter-Glo^®^ Reagent into each well. Mix contents for 2 minutes on an orbital shaker to induce cell lysis. Incubate at room temperature for 10 minutes to stabilize luminescent signal. Record luminescence using EnVision Multilabel Reader (PerkinElmer). Cell viability (CV%) was calculated relative to vehicle (DMSO) treated control wells using following formula: Cell viability (%) = (RLU compound - RLU blank) / (RLU control - RLU blank) * 100%. The IC_50_ values were calculated using GraphPad Prism 6.0 software, fitting to a 4-parameter equation to generate concentration-response curves.

The *in vitro* antitumor assay against HeLa cell was evaluated by the Cell Counting Kit-8 (CCK8, DOJINDO, Japan) assay. The HeLa cells were seeded at a density of 5×10^3^ cells per well into a 96-well microplate in 100 μL of growth medium. Cells were incubated at 37 ^o^C and 5% CO_2_ overnight. Subsequently, 100 μL of the 0.1% DMSO medium solution containing the tested compound in the final concentration from 32 nM to 500 μM was added to the well. The control group was treated with 100 μL of 0.1% DMSO in medium. Cells were incubated at 37 ^o^C and 5% CO_2_ for 72 hours. Then 10 μL CCK8 was added to each well. The plates were incubated at 37 ^o^C for 2 hours, after that the plates were recorded by measuring absorbance at 450 nm with the reference wavelength of 630 nm using an EnVision Multilabel Reader (PerkinElmer). The IC_50_ values were calculated using GraphPad Prism 6.0 software and determined by the concentration causing a half-maximal percent activity.

**4. Characterization of compounds**

*2-phenyl-3-(trifluoromethyl)-4H-thiochromen-4-one (****3a****)*

45.9 mg, 75%; Yellow solid, m.p. 129‑130°C; ^1^H NMR (600 MHz, CDCl_3_) δ 8.54 (dd, *J* = 7.8, 1.2 Hz, 1H), 7.69‑7.67 (m, 1H), 7.61 (t, *J* = 7.2 Hz, 1H), 7.56 (d, *J* = 8.4 Hz, 1H), 7.53-7.43 (m, 5H). ^13^C NMR (150 MHz, CDCl_3_) δ 177.7, 158.4 (q, *J* = 3.0 Hz), 136.1, 135.4, 132.6, 131.6 (q, *J* = 1.5 Hz), 130.5, 129.4, 128.7, 128.6, 128.0 (q, *J* = 1.3 Hz), 125.4, 122.74 (q, *J* = 276 Hz), 122.70 (q, *J* = 27 Hz). ^19^F NMR (565 MHz, CDCl_3_) δ -55.7 (s, 3F). HRMS Calcd for C_16_H_10_F_3_OS [M + H]^+^: m/z 307.0399, Found: 307.0390.

*2-(p-tolyl)-3-(trifluoromethyl)-4H-thiochromen-4-one (****3b****)*

35.2 mg, 55%; Yellow solid, m.p. 109‑110°C; ^1^H NMR (600 MHz, CDCl_3_) δ 8.53 (dd, *J* = 8.4, 1.2 Hz, 1H), 7.68‑7.65 (m, 1H), 7.61‑7.58 (m, 1H), 7.55 (d, *J* = 8.4 Hz, 1H), 7.34 (d, *J* = 7.8 Hz, 2H), 7.28 (d, *J* = 7.8 Hz, 2H), 2.43 (s, 3H). ^13^C NMR (150 MHz, CDCl_3_) δ 177.8, 158.7 (q, *J* = 2.2 Hz), 140.9, 136.2, 132.54, 132.50, 131.6, 129.4, 129.3, 128.6, 127.9 (q, *J* = 1.5 Hz), 125.4, 122.8 (q, *J* = 275 Hz), 122.6 (q, *J* = 26 Hz), 21.6. ^19^F NMR (565 MHz, CDCl_3_) δ -55.7 (s, 3F). HRMS Calcd for C_17_H_12_F_3_OS [M + H]^+^: m/z 321.0555, Found: 321.0542.

*2-(4-ethylphenyl)-3-(trifluoromethyl)-4H-thiochromen-4-one (****3c****)*

52.1 mg, 78%; Yellow solid, m.p. 101‑102°C; ^1^H NMR (600 MHz, CDCl_3_) δ 8.53 (dd, *J* = 8.4, 1.2 Hz, 1H), 7.68‑7.65 (m, 1H), 7.61-7.58 (m, 1H), 7.55 (d, *J* = 7.8 Hz, 1H), 7.36 (d, *J* = 8.4 Hz, 2H), 7.30 (d, *J* = 7.8 Hz, 2H), 2.73 (q, *J* = 7.2 Hz, 2H), 1.29 (t, *J* = 7.2 Hz, 3H). ^13^C NMR (150 MHz, CDCl_3_) δ ^13^C NMR (150 MHz, CDCl_3_) δ 177.8, 158.7 (q, *J* = 2.2 Hz), 147.0, 136.2, 132.7, 132.5, 131.6, 129.4, 128.6, 128.1, 128.0 (q, *J* = 1.5 Hz), 125.4, 122.8 (q, *J* = 276 Hz), 122.5 (q, *J* = 27 Hz), 28.9, 15.3.^19^F NMR (565 MHz, CDCl_3_) δ -55.7 (s, 3F). HRMS Calcd for C_18_H_14_F_3_OS [M + H]^+^: m/z 335.0712 Found: 335.0696.

*2-(4-(tert-butyl)phenyl)-3-(trifluoromethyl)-4H-thiochromen-4-one (****3d****)*

54.3 mg, 75%; Yellow solid, m.p. 101‑102°C; ^1^H NMR (600 MHz, CDCl_3_) δ 8.53 (d, *J* = 8.4 Hz, 1H), 7.68‑7.66 (m, 1H), 7.60 (t, *J* = 7.2 Hz, 1H), 7.55 (d, *J* = 7.8 Hz, 1H), 7.48 (d, *J* = 7.8 Hz, 2H), 7.38 (d, *J* = 7.8 Hz, 2H), 1.37 (s, 9H). ^13^C NMR (150 MHz, CDCl_3_) δ 177.9, 158.8 (q, *J* = 1.5 Hz), 154.0, 136.3, 132.48, 132.47, 131.6, 129.4, 128.6, 127.8, 125.6, 125.4, 122.8 (q, *J* = 274 Hz), 122.5 (q, *J* = 27 Hz), 35.1, 31.3. ^19^F NMR (376 MHz, CDCl_3_) δ -55.7 (s, 3F). HRMS Calcd for C_20_H_18_F_3_OS [M + H]^+^: m/z 363.1025 Found: 363.1016.

*2-(4-methoxyphenyl)-3-(trifluoromethyl)-4H-thiochromen-4-one (****3e****)*

46.4 mg, 69%; Yellow solid, m.p. 122‑123°C; ^1^H NMR (600 MHz, CDCl_3_) δ 8.52 (d, *J* = 7.8 Hz, 1H), 7.68‑7.65 (m, 1H), 7.59 (t, *J* = 7.8 Hz, 1H), 7.55 (d, *J* = 7.8 Hz, 1H), 7.40 (d, *J* = 8.4 Hz, 2H), 6.99 (d, *J* = 8.4 Hz, 2H), 3.88 (s, 3H). ^13^C NMR (150 MHz, CDCl_3_) δ 178.0, 161.5, 158.4 (q, *J* = 3.0 Hz), 136.2, 132.5, 131.7, 129.7 (q, *J* = 1.5 Hz), 129.4, 128.6, 127.6, 125.4, 122.9 (q, *J* = 276 Hz), 122.5 (q, *J* = 27 Hz), 114.1, 55.6.^19^F NMR (565 MHz, CDCl_3_) δ -55.6 (s, 3F). HRMS Calcd for C_17_H_12_F_3_O_2_S [M + H]^+^: m/z 337.0505 Found: 337.0494.

*2-(m-tolyl)-3-(trifluoromethyl)-4H-thiochromen-4-one (****3f****)*

44.8 mg, 70%; Yellow solid, m.p. 54‑55°C; ^1^H NMR (600 MHz, CDCl_3_) δ 8.54 (dd, *J* = 8.4, 1.2 Hz, 1H), 7.69‑7.66 (m, 1H), 7.61‑7.59 (m, 1H), 7.55 (d, *J* = 7.8 Hz, 1H), 7.36 (t, *J* = 7.8 Hz, 1H), 7.32 (d, *J* = 7.8 Hz, 1H), 7.25-7.23 (m, 2H), 2.43 (s, 3H). ^13^C NMR (150 MHz, CDCl_3_) δ 177.7, 158.7 (q, *J* = 3.0 Hz), 138.5, 136.1, 135.3, 132.5, 131.6, 131.2, 129.4, 128.7, 128.49, 128.47, 125.4, 125.2, 122.8 (q, *J* = 275 Hz), 122.6 (q, *J* = 27 Hz), 21.5. ^19^F NMR (376 MHz, CDCl_3_) δ -55.8 (s, 3F). HRMS Calcd for C_17_H_12_F_3_OS [M + H]^+^: m/z 321.0555 Found:321.0519.

*2-(4-fluorophenyl)-3-(trifluoromethyl)-4H-thiochromen-4-one (****3g****)*

52.5 mg, 81%; Yellow solid, m.p. 105‑106°C; ^1^H NMR (600 MHz, CDCl_3_) δ 8.53 (dd, *J* = 7.8, 0.6 Hz, 1H), 7.70‑7.67 (m, 1H), 7.62-7.60 (m, 1H), 7.56 (d, *J* = 8.4 Hz, 1H), 7.45‑7.43 (m, 2H), 7.19‑7.16 (m, 2H). ^13^C NMR (150 MHz, CDCl_3_) δ 177.6, 164.0 (d, *J* = 249 Hz), 157.2 (q, *J* = 3.0 Hz), 135.8, 132.7, 131.6, 131.3 (d, *J* = 3.0 Hz), 130.1 (dd, *J* = 9.0, 1.5 Hz), 129.4, 128.8, 125.4, 123.0 (q, *J* = 27 Hz), 122.7 (q, *J* = 274.5 Hz), 116.0 (d, *J* = 22.5 Hz). ^19^F NMR (565 MHz, CDCl_3_) δ -55.6 (s, 3F), -109.5 (s, 1F). HRMS Calcd for C_16_H_9_F_4_OS [M + H]^+^: m/z 325.0305 Found: 325.0277.

*2-(4-chlorophenyl)-3-(trifluoromethyl)-4H-thiochromen-4-one (****3h****)*

40.8 mg, 60%; Yellow solid, m.p. 119‑120°C; ^1^H NMR (400 MHz, CDCl_3_) δ 8.53 (d, *J* = 8.0 Hz, 1H), 7.71-7.67 (m, 1H), 7.61 (t, *J* = 7.6 Hz, 1H), 7.56 (d, *J* = 8.0 Hz, 1H), 7.46 (d, *J* = 8.4 Hz, 2H), 7.38 (d, *J* = 8.4 Hz, 2H). ^13^C NMR (150 MHz, CDCl_3_) δ 177.5, 156.9 (q, *J* = 3.0 Hz), 136.9, 135.8, 133.7, 132.7, 131.5, 129.43, 129.36 (q, *J* = 1.5 Hz), 129.0, 128.9, 125.5, 122.9 (q, *J* = 27 Hz), 122.6 (q, *J* = 276 Hz). ^19^F NMR (565 MHz, CDCl_3_) δ -55.6 (s, 3F). HRMS Calcd for C_16_H_9_ClF_3_OS [M + H]^+^: m/z 341.0009 Found: 340.9990.

*2-(4-bromophenyl)-3-(trifluoromethyl)-4H-thiochromen-4-one (****3i****)*

66.2 mg, 86%; Yellow solid, m.p. 132‑133°C; ^1^H NMR (400 MHz, CDCl_3_) δ 8.53 (d, *J* = 8.0 Hz, 1H), 7.71-7.67 (m, 1H), 7.63-7.60 (m, 3H), 7.56 (d, *J* = 8.0 Hz, 1H), 7.32 (d, *J* = 8.0 Hz, 2H). ^13^C NMR (150 MHz, CDCl_3_) δ 177.5, 156.9 (q, *J* = 1.5 Hz), 135.7, 134.2, 132.7, 132.0, 131.5, 129.55 (q, *J* = 1.5 Hz), 129.46, 128.9, 125.5, 125.1, 122.9 (q, *J* = 27 Hz), 122.6 (q, *J* = 276 Hz). ^19^F NMR (565 MHz, CDCl_3_) δ -55.6 (s, 3F). HRMS Calcd for C_16_H_9_BrF_3_OS [M + H]^+^: m/z 384.9504 Found: 384.9496.

*2-(3-fluorophenyl)-3-(trifluoromethyl)-4H-thiochromen-4-one (****3j****)*

30.5 mg, 47%; Yellow solid, m.p. 113‑114°C; 1H NMR (600 MHz, CDCl3) δ 8.54 (dd, *J* = 8.4, 1.2 Hz, 1H), 7.71‑7.68 (m, 1H), 7.63‑7.60 (m, 1H), 7.56 (d, *J* = 7.8 Hz, 1H), 7.48-7.44 (m, 1H), 7.23‑7.20 (m, 2H), 7.18‑7.16 (m, 1H). ^13^C NMR (150 MHz, CDCl_3_) δ 177.4, 162.3 (d, *J* = 247.5 Hz), 156.6 (q, *J* = 3.0 Hz), 137.0 (d, *J* = 7.5 Hz), 135.7, 132.7, 131.5, 130.5 (d, *J* = 9.0 Hz), 129.4, 128.9, 125.5, 123.9 (q, *J* = 1.5 Hz), 123.0 (q, *J* = 27 Hz), 122.6 (q, *J* = 274.5 Hz), 117.5 (d, *J* = 21 Hz), 115.4 (dq, *J* = 22.5, 1.5 Hz). ^19^F NMR (565 MHz, CDCl_3_) δ -55.8 (s, 3F), -111.5 (s, 1F). HRMS Calcd for C_16_H_9_F_4_OS [M + H]^+^: m/z 325.0305 Found: 325.0298.

*2-(2-bromophenyl)-3-(trifluoromethyl)-4H-thiochromen-4-one (****3k****)*

57.7 mg, 75%; Yellow solid, m.p. 132‑133°C; ^1^H NMR (600 MHz, CDCl_3_) δ 8.58 (d, *J* = 7.8 Hz, 1H), 7.71-7.68 (m, 2H), 7.62 (t, *J* = 7.8 Hz, 1H), 7.57 (d, *J* = 7.8 Hz, 1H), 7.45-7.42 (m, 1H), 7.37-7.34 (m, 2H). ^13^C NMR (150 MHz, CDCl_3_) δ 177.1, 156.3 (q, *J* = 3.0 Hz), 136.1, 135.9, 133.2, 132.7, 131.43, 131.38, 129.5, 129.4, 128.9, 127.5, 125.6, 124.0 (q, *J* = 27 Hz), 122.5 (q, *J* = 276 Hz), 121.8 (q, *J* = 3.0 Hz). ^19^F NMR (376 MHz, CDCl_3_) δ -58.4 (s, 3F). HRMS Calcd for C_16_H_9_BrF_3_OS [M + H]^+^: m/z 384.9504 Found: 384.9495.

*3-(trifluoromethyl)-2-(4-(trifluoromethyl)phenyl)-4H-thiochromen-4-one (****3l****)*

65.8 mg, 88%; Yellow solid, m.p. 127‑128°C; ^1^H NMR (400 MHz, CDCl_3_) δ 8.55 (d, *J* = 8.0 Hz, 1H), 7.78‑7.69 (m, 3H), 7.63 (t, *J* = 7.6 Hz, 1H), 7.58-7.56 (m, 3H). ^13^C NMR (150 MHz, CDCl_3_) δ 177.2, 156.4 (q, *J* = 3.0 Hz), 138.8, 135.5, 132.8, 132.5 (q, *J* = 33 Hz), 131.4 (q, *J* = 0.9 Hz), 129.5, 129.1, 128.5 (q, *J* = 1.5 Hz), 125.7 (q, *J* = 3.0 Hz), 125.5, 123.7 (q, *J* = 270 Hz), 123.1 (q, *J* = 27 Hz), 122.5 (q, *J* = 276 Hz). ^19^F NMR (565 MHz, CDCl_3_) δ -55.7 (s, 3F), -62.9 (s, 3F). HRMS Calcd for C_17_H_9_F_6_OS [M + H]^+^: m/z 375.0273 Found: 375.0259.

*4-(4-oxo-3-(trifluoromethyl)-4H-thiochromen-2-yl)benzonitrile (****3m****)*

30.5 mg, 46%; Yellow solid, m.p. 100-101°C; ^1^H NMR (600 MHz, CDCl_3_) δ 8.54 (dd, *J* = 7.8, 1.2 Hz, 1H), 7.79 (d, *J* = 8.4 Hz, 2H), 7.73‑7.70 (m, 1H), 7.66‑7.63 (m, 1H), 7.58-7.56 (m, 3H). ^13^C NMR (150 MHz, CDCl_3_) δ 177.1, 155.6 (q, *J* = 3.0 Hz), 139.6, 135.3, 133.0, 132.5, 131.4, 129.5, 129.2, 128.8 (q, *J* = 1.5 Hz), 125.6, 123.2 (q, *J* = 27 Hz), 122.5 (q, *J* = 276 Hz), 117.9, 114.5. ^19^F NMR (565 MHz, CDCl_3_) δ -55.6 (s, 3F). HRMS Calcd for C_17_H_9_F_3_NOS [M + H]^+^: m/z 332.0351 Found: 332.0345.

*2-(pyridin-3-yl)-3-(trifluoromethyl)-4H-thiochromen-4-one (****3n****)*

31.3 mg, 51%; Yellow solid, m.p. 65-66 °C; ^1^H NMR (600 MHz, CDCl_3_) δ 8.76 (dd, *J* = 4.8, 1.2 Hz, 1H), 8.70 (d, *J* = 1.8 Hz, 1H), 8.54 (dd, *J* = 8.4, 1.2 Hz, 1H), 7.78‑7.76 (m, 1H), 7.72-7.69 (m, 1H), 7.65‑7.62 (m, 1H), 7.58 (dd, *J* = 7.8, 0.6 Hz, 1H), 7.45-7.43 (m, 1H). ^13^C NMR (150 MHz, CDCl_3_) δ 177.2, 154.3, 151.5, 147.9, 135.5, 135.4 (q, *J* = 3.0 Hz), 132.8, 131.6, 131.4, 129.5, 129.1, 125.5, 123.6 (q, *J* = 27 Hz), 123.3, 122.6 (q, *J* = 274.5 Hz). ^19^F NMR (565 MHz, CDCl_3_) δ -55.4 (s, 3F). HRMS Calcd for C_15_H_9_F_3_NOS [M + H]^+^: m/z 308.0351 Found: 308.0348.

*2-(naphthalen-2-yl)-3-(trifluoromethyl)-4H-thiochromen-4-one (****3o****)*

23.5 mg, 33%; Yellow solid, m.p. 51‑52°C; ^1^H NMR (400 MHz, CDCl_3_) δ 8.57 (dd, *J* = 8.0, 1.2 Hz, 1H), 7.96‑7.91 (m, 4H), 7.72‑7.68 (m, 1H), 7.65-7.57 (m, 4H), 7.52 (dd, *J* = 8.4, 2.0 Hz, 1H). ^13^C NMR (150 MHz, CDCl_3_) δ 177.7, 158.4 (q, *J* = 1.5 Hz), 136.2, 133.9, 132.8, 132.6, 132.1, 131.6, 129.5, 128.8, 128.7, 128.5, 128.1, 127.8, 127.7, 127.3, 125.5, 125.3, 122.9 (q, *J* = 27 Hz), 122.8 (q, *J* = 276 Hz).^19^F NMR (565 MHz, CDCl_3_) δ -55.7 (s, 3F). HRMS Calcd for C_20_H_12_F_3_OS [M + H]^+^: m/z 357.0555 Found: 357.0545.

*2-hexyl-3-(trifluoromethyl)-4H-thiochromen-4-one (****3p****)*

39.6 mg, 63%; Colorless oil; ^1^H NMR (400 MHz, CDCl_3_) δ 8.46 (dd, *J* = 8.0, 0.8 Hz, 1H), 7.65‑7.61 (m, 1H), 7.67‑7.51 (m, 2H), 2.90‑2.86 (m, 2H), 1.80-1.72 (m, 2H), 1.48-1.41 (m, 2H), 1.34-1.31 (m, 4H), 0.90 (t, *J* = 6.8 Hz, 3H). ^13^C NMR (100 MHz, CDCl_3_) δ 177.6, 161.2 (q, *J* = 1.0 Hz), 135.6, 132.2, 131.3, 129.4, 128.4, 125.3, 123.7 (q, *J* = 276 Hz), 122.6 (q, *J* = 27 Hz), 37.0 (q, *J* = 2.0 Hz), 31.6 (q, *J* = 1.0 Hz), 31.4, 29.4, 22.6, 14.1.^19^F NMR (565 MHz, CDCl_3_) δ -55.4 (s, 3F). HRMS Calcd for C_16_H_18_F_3_OS [M + H]^+^: m/z 315.1025 Found: 315.1028.

*3-(perfluoroethyl)-2-phenyl-4H-thiochromen-4-one* *(****3q****)*

52.0 mg, 73%; Yellow solid, m.p. 116-117 °C; ^1^H NMR (400 MHz, CDCl_3_) δ 8.54 (dd, *J* = 8.0, 1.2 Hz, 1H), 7.70‑7.66 (m, 1H), 7.63‑7.58 (m, 1H), 7.53 (d, *J* = 8.0 Hz, 1H), 7.48‑7.42 (m, 3H), 7.37 (d, *J* = 6.8 Hz, 2H). ^13^C NMR (150 MHz, CDCl_3_) δ 177.6, 160.7 (t, *J* = 3.0 Hz), 136.0, 135.3, 132.6, 131.4, 129.9, 129.4, 128.8, 128.1, 127.8, 125.2, 122.2-112.2 (m). ^19^F NMR (376 MHz, CDCl_3_) δ -80.1 (s, 3F), -102.6 (s, 2F). HRMS Calcd for C_17_H_10_F_5_OS [M + H]^+^: m/z 357.0367 Found: 357.0389.

*3-(perfluorobutyl)-2-phenyl-4H-thiochromen-4-one (****3r****)*

36.5 mg, 40%; Yellow solid, m.p. 82-83°C; ^1^H NMR (600 MHz, CDCl_3_) δ 8.54 (d, *J* = 8.4 Hz, 1H), 7.68 (t, *J* = 7.8 Hz, 1H), 7.61 (t, *J* = 7.8 Hz, 1H), 7.53 (d, *J* = 7.8 Hz, 1H), 7.49-7.42 (m, 3H), 7.37 (d, *J* = 7.2 Hz, 2H). ^13^C NMR (150 MHz, CDCl_3_) δ 177.7, 160.9 (t, *J* = 3.0 Hz), 136.0, 135.1, 132.7, 131.5, 129.9, 129.5, 128.9, 128.1, 127.9, 125.1, 122.3-110.6 (m). ^19^F NMR (565 MHz, CDCl_3_) δ -80.7 (t, *J* = 11.3 Hz, 3F), -99.3 (t, *J* = 17.0 Hz, 2F), -117.6 (m, 2F), -126.2 (m, 2F). HRMS Calcd for C_19_H_10_F_9_OS [M + H]^+^: m/z 457.0303 Found: 457.0308.

*3-(perfluorohexyl)-2-phenyl-4H-thiochromen-4-one (****3s****)*

46.7 mg, 42%; Yellow solid, m.p. 76-77 °C; ^1^H NMR (400 MHz, CDCl_3_) δ 8.54 (dd, *J* = 8.0, 1.2 Hz, 1H), 7.70‑7.66 (m, 1H), 7.63‑7.59 (m, 1H), 7.53 (dd, *J* = 8.0, 0.8 Hz, 1H), 7.48‑7.42 (m, 3H), 7.37 (d, *J* = 7.2 Hz, 2H). ^13^C NMR (150 MHz, CDCl_3_) δ 177.7, 160.9 (t, *J* = 3.0 Hz), 136.0, 135.1, 132.7, 131.5, 129.9, 129.5, 128.9, 128.1, 127.9, 125.1, 122.4 (t, *J* = 18 Hz), 119.2-109.1 (m). ^19^F NMR (376 MHz, CDCl_3_) δ -80.8 (t, *J* = 11.3 Hz, 3F), -99.2 (m, 2F), -116.8 (m, 2F), -122.0 (m, 2F), -122.5 (m, 2F), -126.1 (m, 2F). HRMS Calcd for C_21_H_10_F_13_OS [M + H]^+^: m/z 557.0239 Found: 557.0253.

*3-(perfluorooctyl)-2-phenyl-4H-thiochromen-4-one (****3t****)*

56.4 mg, 43%; Yellow solid, m.p. 68-69 °C; ^1^H NMR (600 MHz, CDCl_3_) δ 8.54 (d, *J* = 8.4 Hz, 1H), 7.69-7.66 (m, 1H), 7.62-7.59 (m, 1H), 7.53 (d, *J* = 7.8 Hz, 1H), 7.49-7.42 (m, 3H), 7.37 (d, *J* = 7.8 Hz, 2H). ^13^C NMR (150 MHz, CDCl_3_) δ 177.7, 160.9 (t, *J* = 3.0 Hz), 136.0, 135.2, 132.6, 131.5, 129.9, 129.5, 128.9, 128.1, 127.9, 125.1, 122.4 (t, *J* = 19.5 Hz), 119.2-107.9 (m). ^19^F NMR (376 MHz, CDCl_3_) δ -80.8 (t, *J* = 11.3 Hz, 3F), -99.2 (m, 2F), -116.7 (m, 2F), -121.5 (m, 2F), -121.8 (m, 2F), -121.9 (m, 2F), -122.7 (m, 2F), -126.1 (m, 2F). HRMS Calcd for C_23_H_10_F_17_OS [M + H]^+^: m/z 657.0175 Found:657.0171.

*3-(perfluoroethyl)-2-(p-tolyl)-4H-thiochromen-4-one (****3u****)*

62.2 mg, 84%; Yellow solid, m.p. 122-123 °C; ^1^H NMR (400 MHz, CDCl_3_) δ 8.54 (dd, *J* = 8.0, 1.2 Hz, 1H), 7.70‑7.65 (m, 1H), 7.62‑7.58 (m, 1H), 7.53 (dd, *J* = 8.0, 0.8 Hz, 1H), 7.28‑7.24 (m, 4H), 2.43 (s, 3H). ^13^C NMR (150 MHz, CDCl_3_) δ 177.7, 161.0 (t, *J* = 3.0 Hz), 140.1, 136.1, 132.6, 132.4, 131.5, 129.4, 128.8, 128.7, 127.7, 125.1, 122.2-112.3 (m), 21.5. ^19^F NMR (376 MHz, CDCl_3_) δ -80.0 (s, 3F), -102.6 (s, 2F). HRMS Calcd for C_18_H_12_F_5_OS [M + H]^+^: m/z 371.0524 Found: 371.0530.

*3-(perfluorobutyl)-2-(p-tolyl)-4H-thiochromen-4-one (****3v****)*

68.6 mg, 73%; Yellow solid, m.p. 61-62 °C; ^1^H NMR (600 MHz, CDCl_3_) δ 8.53 (d, *J* = 8.4 Hz, 1H), 7.68-7.66 (m, 1H), 7.61-7.58 (m, 1H), 7.52 (d, *J* = 7.8 Hz, 1H), 7.25-7.23 (m, 4H), 2.43 (s, 3H). ^13^C NMR (150 MHz, CDCl_3_) δ 177.8, 161.2, 140.1, 136.2, 132.6, 132.3, 131.5, 129.4, 128.8, 127.8, 125.1, 122.4-111.5 (m), 21.5. ^19^F NMR (565 MHz, CDCl_3_) δ -80.7 (t, *J* = 11.3 Hz, 3F), -99.3 (t, *J* = 17.0 Hz, 2F), -117.5 (m, 2F), -126.2 (m, 2F). HRMS Calcd for C_20_H_12_F_9_OS [M + H]^+^: m/z 471.0460 Found: 471.0448.

*2-(4-ethylphenyl)-3-(perfluorohexyl)-4H-thiochromen-4-one (****3w****)*

64.2 mg, 55%; Yellow solid, m.p. 83-84 °C; ^1^H NMR (600 MHz, CDCl_3_) δ 8.53 (d, *J* = 7.8 Hz, 1H), 7.68-7.65 (m, 1H), 7.59 (t, *J* = 7.8 Hz, 1H), 7.51 (d, *J* = 7.8 Hz, 1H), 7.28-7.25 (m, 4H), 2.72 (q, *J* = 7.8 Hz, 2H), 1.29 (t, *J* = 7.8 Hz, 3H). ^13^C NMR (150 MHz, CDCl_3_) δ 177.8, 161.3 (t, *J* = 3.0 Hz), 146.3, 136.2, 132.6, 132.5, 131.6, 129.4, 128.8, 127.9, 127.5, 125.1, 122.4 (t, *J* = 19.5 Hz), 119.2-106.8 (m), 28.8, 15.3. ^19^F NMR (565 MHz, CDCl_3_) δ -80.8 (t, *J* = 11.3 Hz, 3F), -99.1 (t, *J* = 17.0 Hz, 2F), -116.7 (t, *J* = 17.0 Hz, 2F), -121.9 (m, 2F), -122.5 (m, 2F), -126.1 (m, 2F). HRMS Calcd for C_23_H_14_F_13_OS [M + H]^+^: m/z 585.0552 Found:585.0528.

*2-(4-methoxyphenyl)-3-(perfluorooctyl)-4H-thiochromen-4-one (****3x****)*

74.1 mg, 54%; Yellow solid, m.p. 72-73 °C; ^1^H NMR (600 MHz, CDCl_3_) δ 8.53 (d, *J* = 8.4 Hz, 1H), 7.66 (t, *J* = 7.2 Hz, 1H), 7.59 (t, *J* = 7.8 Hz, 1H), 7.52 (d, *J* = 8.4 Hz, 1H), 7.30 (d, *J* = 8.4 Hz, 2H), 6.95 (d, *J* = 7.8 Hz, 2H), 3.87 (s, 3H). ^13^C NMR (150 MHz, CDCl_3_) δ 177.9, 161.0 (t, *J* = 3.0 Hz), 160.9, 136.2, 132.6, 131.6, 129.43, 129.40, 128.8, 127.3, 125.1, 122.6 (t, *J* = 19.5 Hz), 119.5-115.8 (m), 113.6, 113.3-108.4 (m), 55.5. ^19^F NMR (376 MHz, CDCl_3_) δ -80.8 (t, *J* = 9.8 Hz, 3F), -99.1 (m, 2F), -116.6 (m, 2F), -121.5 (m, 2F), -121.7 (m, 2F), -121.8 (m, 2F), -122.7 (m, 2F), -126.1 (m, 2F). HRMS Calcd for C_24_H_12_F_17_O_2_S [M + H]^+^: m/z 687.0281 Found:687.0268.

*2-(4-fluorophenyl)-3-(perfluorooctyl)-4H-thiochromen-4-one (****3y****)*

55.3 mg, 41%; Yellow solid, m.p. 91-92 °C; ^1^H NMR (600 MHz, CDCl_3_) δ 8.53 (d, *J* = 8.4 Hz, 1H), 7.69 (t, *J* = 7.8 Hz, 1H), 7.61 (t, *J* = 7.8 Hz, 1H), 7.53 (d, *J* = 7.8 Hz, 1H), 7.37-7.35 (m, 2H), 7.15-7.12 (m, 2H). ^13^C NMR (150 MHz, CDCl_3_) δ 177.6, 163.6 (d, *J* = 249 Hz), 159.8 (t, *J* = 3.0 Hz), 135.8, 132.7, 131.5, 131.0, 130.0 (d, *J* = 7.5 Hz), 129.5, 129.0, 125.1, 122.8 (t, *J* = 18 Hz), 120.2-115.7 (m), 115.4 (d, *J* = 22.5 Hz), 113.3-108.7 (m). ^19^F NMR (565 MHz, CDCl_3_) δ -80.8 (t, *J* = 9.6 Hz, 3F), -99.2 (t, *J* = 13.6 Hz, 2F), -110.5 (s, 1F), -116.8 (m, 2F), -121.5 (s, 2F), -121.7 (s, 2F), -121.9 (s, 2F), -122.7 (s, 2F), -126.1 (m, 2F). HRMS Calcd for C_23_H_9_F_18_OS [M + H]^+^: m/z 675.0081 Found:675.0094.

*5-chloro-1,3-dimethyl-3-(2,2,2-trifluoroethyl)indolin-2-one (****5****)*^1^

32.1 mg, 58%; Yellow solid, m.p. 67-68 °C; ^1^H NMR (400 MHz, CDCl_3_) δ 7.29 (dd, *J* = 8.4, 2.0 Hz, 1H), 7.24 (d, *J* = 2.0 Hz, 1H), 6.80 (d, *J* = 8.4 Hz, 1H), 3.22 (s, 3H), 2.88-2.76 (m, 1H), 2.68-2.57 (m, 1H), 1.40 (s, 3H). ^13^C NMR (150 MHz, CDCl_3_) δ 178.1, 141.6, 132.8, 128.7, 128.2, 125.2 (q, *J* = 276 Hz), 124.2, 109.5, 44.7 (q, *J* = 1.5 Hz), 40.7 (q, *J* = 28.5 Hz), 26.7, 25.1. ^19^F NMR (376 MHz, CDCl_3_) δ -62.0 (s, 3F). HRMS Calcd for C_12_H_12_ClF_3_NO [M + H]^+^: m/z 278.0554 Found:278.0545.

*1,3-dimethyl-3-(2,2,2-trifluoroethyl)quinoline-2,4(1H,3H)-dione (****7****)*^2^

47.7 mg, 88%; Yellow solid, m.p. 75-76 °C; ^1^H NMR (400 MHz, CDCl_3_) δ 8.09 (dd, *J* = 8.0, 1.6 Hz, 1H), 7.70‑7.66 (m, 1H), 7.24-7.20 (m, 2H), 3.51 (s, 3H), 3.07 (q, *J* = 10.4 Hz, 2H), 1.50 (s, 3H). ^13^C NMR (100 MHz, CDCl_3_) δ 194.5, 171.8, 143.3, 136.8, 128.8, 125.4 (q, *J* = 277 Hz), 123.6, 119.2, 115.2, 52.5 (q, *J* = 3.0 Hz), 40.7 (q, *J* = 29 Hz), 30.1, 27.1. ^19^F NMR (376 MHz, CDCl_3_) δ -60.7 (s, 3F). HRMS Calcd for C_13_H_13_F_3_NO_2_ [M + H]^+^: m/z 272.0893 Found:272.0879.

**4. NMR copies of products**

Figure S2. NMR spectra copies of product **3a**

Figure S3. NMR spectra copies of product **3b**

Figure S4. NMR spectra copies of product **3c**

Figure S5. NMR spectra copies of product **3d**

Figure S6. NMR spectra copies of product **3e**

Figure S7. NMR spectra copies of product **3f**

Figure S8. NMR spectra copies of product **3g**

Figure S9. NMR spectra copies of product **3h**

Figure S10. NMR spectra copies of product **3i**

Figure S11. NMR spectra copies of product **3j**

Figure S12. NMR spectra copies of product **3k**

Figure S13. NMR spectra copies of product **3l**

Figure S14. NMR spectra copies of product **3m**

Figure S15. NMR spectra copies of product **3n**

Figure S16. NMR spectra copies of product **3o**

Figure S17. NMR spectra copies of product **3p**

Figure S18. NMR spectra copies of product **3q**

Figure S19. NMR spectra copies of product **3r**

Figure S20. NMR spectra copies of product **3s**

Figure S21. NMR spectra copies of product **3t**

Figure S22. NMR spectra copies of product **3u**

Figure S23. NMR spectra copies of product **3v**

Figure S24. NMR spectra copies of product **3w**

Figure S25. NMR spectra copies of product **3x**

Figure S26. NMR spectra copies of product **3y**

Figure S27. NMR spectra copies of product **5**

Figure S28. NMR spectra copies of product **7**

**5. X-ray crystallographic data for 3a**

| **Table 1 Crystal data and structure refinement for 3a.** | |
| --- | --- |
| Identification code | 2152392 |
| Empirical formula | C_16_H_9_F_3_OS |
| Formula weight | 306.29 |
| Temperature/K | 291.59(10) |
| Crystal system | monoclinic |
| Space group | P2_1_/c |
| a/Å | 13.4206(6) |
| b/Å | 11.7063(6) |
| c/Å | 8.6253(4) |
| α/° | 90 |
| β/° | 91.577(5) |
| γ/° | 90 |
| Volume/Å^3^ | 1354.58(12) |
| Z | 4 |
| ρ_calc_g/cm^3^ | 1.502 |
| μ/mm^‑1^ | 0.268 |
| F(000) | 624.0 |
| Crystal size/mm^3^ | 0.1 × 0.05 × 0.05 |
| Radiation | Mo Kα (λ = 0.71073) |
| 2Θ range for data collection/° | 6.668 to 58.064 |
| Index ranges | -12 ≤ h ≤ 18, -14 ≤ k ≤ 15, -10 ≤ l ≤ 11 |
| Reflections collected | 9531 |
| Independent reflections | 3173 [R_int_ = 0.0297, R_sigma_ = 0.0372] |
| Data/restraints/parameters | 3173/0/190 |
| Goodness-of-fit on F^2^ | 1.021 |
| Final R indexes [I>=2σ (I)] | R_1_ = 0.0520, wR_2_ = 0.1110 |
| Final R indexes [all data] | R_1_ = 0.0808, wR_2_ = 0.1270 |
| Largest diff. peak/hole / e Å^-3^ | 0.24/-0.21 |

**6. References**

1. Zhang, L. Z., Li, Z. J., Liu, Z. Q. A Free-Radical Cascade Trifluoromethylation/Cyclization of N-Arylmethacrylamides and Enynes with Sodium Trifluoromethanesulfinate and Iodine Pentoxide. *Org*. *Lett*. **2014**, *16*, 3688-3691.

2. Fu, H., Wang, S. S., Li, Y. M. Copper-Mediated Oxidative Radical Addition/Cyclization Cascade: Synthesis of Trifluoromethylated and Sulfonated Quinoline-2,4(1H,3H)-diones. *Adv*. *Synth*. *Catal*. **2016**, *358*, 3616-3626.
